# Individual and institutional factors associated with functional disability in nursing home residents: An observational study with multilevel analysis

**DOI:** 10.1371/journal.pone.0183945

**Published:** 2017-08-28

**Authors:** Ramón Serrano-Urrea, Virgilio Gómez-Rubio, Domingo Palacios-Ceña, César Fernández-de-las-Peñas, María José García-Meseguer

**Affiliations:** 1 Department of Mathematics, Faculty of Computer Science Engineering, University of Castilla-La Mancha, Albacete, Spain; 2 Department of Mathematics, Faculty of Industrial Engineering, University of Castilla-La Mancha, Albacete, Spain; 3 Department of Physical Therapy, Occupational Therapy, Rehabilitation and Physical Medicine, Rey Juan Carlos University, Alcorcón, Madrid, Spain; 4 Department of Nursing and Physiotherapy, Faculty of Nursing, University of Castilla-La Mancha, Albacete, Spain; Indiana University Purdue University at Indianapolis, UNITED STATES

## Abstract

**Background:**

High prevalence of functional limitations has been previously observed in nursing homes. Disability may depend not only on the characteristics of the residents but also on the facility characteristics. The aims of this study were: 1, to describe the prevalence of functional disability in older people living in Spanish nursing homes; and 2, to analyze the relationships between individual and nursing home characteristics and residents’ functional disability.

**Methods:**

A cross-sectional study with data collected from 895 residents in 34 nursing homes in the province of Albacete (Spain) was conducted. Functional status was assessed by the Barthel Index. Taking into account both levels of data (individual and institutional characteristics) we resorted to a multilevel analysis in order to take different sources of variability in the data.

**Results:**

The prevalence of functional disability of the total sample was 79.8%. The best fitting multilevel model showed that female gender, older age, negative self-perception of health, and living in private nursing homes were factors significantly associated with functional disability. After separating individual and institutional effects, the institutions showed significant differences.

**Conclusions:**

In line with previous findings, our study found high levels of functional dependence among institutionalized elders. Gender, age, self-perception of health, and institution ownership were associated with functional status. Disentangling individual and institutional effects by means of multilevel models can help evaluate the quality of the residences.

## Introduction

The United Nations reported the number of older persons (≥60 years) in the world is projected to grow by 56%, from 901 million to more than 1.4 billion between 2015 and 2030 [1]. Currently, in Europe, there are 176.5 millions of older people (19.6% total European population) [1]. The ageing process is increasing in Europe and in North America, where more than one of five people was aged 60 or over in 2015, but it is growing rapidly in the other worldwide regions as well [1]. In fact, all countries are expected to see a substantial growth in the number of older persons between 2015 and 2030, and that growth will be faster in the developing regions than in the developed regions [1]. In Spain, the National Statistic Institute [2] reported that in 1 January 2016 there were 8,657,705 older people (65 and over), 18.4% of the total population (46,557,008). In addition, it is estimated that in 2066, there will be more than 14 million elderly people (34.6% of the total population) in Spain [2].

Ageing of society in developed countries represents a real challenge involving sustainability issues which need to be addressed [3]. Home care services and living in nursing homes are resources that society provide to elder people who lose their ability to live independently [3]. For most people, living in a nursing home is a permanent and irreversible situation, being the autonomy loss a factor associated with greater dependence [4] and one of the main factors to leave their own home [5]. In fact, the ability to manage Basic Activities of Daily Living (ADL) is a significant predictor for being housebound, placement in a nursing home and death [6–8].

The World Health Organization International Classification of Functioning, Disability and Health provides a framework for measuring functional levels including environmental and personal factors and recognize subjects’ functioning as a complex interaction of different areas [9]. Therefore, it is important to understand factors associated with functional disability [10] to develop prevention and health care programs [11–16] and increase quality of life [17,18].

The effect of ageing on functional decline, dependence and frailty has been previously studied [6,7,19–21]. In fact, associations between ADL and chemical and anthropometric parameters in older adults have been found [22–25]. Further, different socioeconomic and environmental factors have been also found to be associated with increased dependence [20,21].

Nursing home features such as characteristics of the building, capacity or type of funding could influence, directly or indirectly, residents’ functional ability [26–28]. In Spain, nursing homes are one of the main social care resources available for older people [29]. Admissions are usually related to a need for long-term care, caregiver burnout and limitation or absence of financial resources [30]. Among other objectives, nursing homes in Spain aim to provide a pleasant environment and a safe place to live, to enhance the capabilities of the residents, to prevent disability and loneliness, and to promote an environment where the resident is able to live a life with respect, dignity and autonomy [29,30]. However, Spanish long-term care services have been influenced by familiarism and have traditionally been provided to the majority of the elderly on an informal basis and, on a private, for-profit basis [31,32]. Currently, older people with disability live in a care context where long-term care services based on profit and non-profit (public and private) coexist [31]. Further, the organizational models of care of nursing homes can influence the autonomy, the dependency and coverage of needs of the residents [33,34]. In Spain, organizational models can vary depending on the ownership of the nursing home, and present models from paternalistic management to autonomy models where the preferences of the resident are the center of care [34,35]. In the last years, the increasing in the number of nursing home beds has been particularly important in Spain. During the period 2000–2013, Spain reported one of the highest increases of nursing home beds among European countries with an average annual increase of 3.7 nursing home beds per 1000 population aged 65 and over [36]. The total number of Spanish nursing homes in 2015 was 5340 (3803 private, 1510 public) [37].

Nevertheless, despite the high levels of functional dependence found in institutionalized elderly, only few relevant studies have considered nursing home features as potential factors influencing disability, and the evaluation of both individual and facility-level factors, which may affect functional decline, is suggested in a recent review of the literature [26]. There is a considerable evidence on the influence of specific design features, the facility size or its location on different problems associated with age such as malnutrition [38,39]. No study performed with residents from Albacete has addressed the influence of both individual and nursing home characteristics on functional disability. To the best of the authors’ knowledge, in Albacete, only a few relevant surveys have addressed the study of frailty in nursing homes, but they did not study the influence of nursing home features and did not consider all institutions in the province [8]. An in-depth exploration about individual characteristics of residents and features of the nursing homes on functional disability is shown in the current study.

Therefore, the aims of this paper were: 1, to describe the prevalence of functional disability in elderly people living in nursing homes in the province of Albacete (Spain); and 2, to analyze the relationship between individual and nursing home characteristics and resident’ functional disability.

## Methods

### Study design

A cross-sectional study following STROBE guidelines [40] with data collected from the elderly living in nursing homes all over the province of Albacete (Spain) was conducted.

### Settings

With the exception of one, all the nursing homes in the province agreed to take part in the survey. Data were collected in 34 out of the 37 institutions in the province during the last months of 2009 and 2010 by three trained nurses. Individual assessments were conducted by interviewing the residents meeting inclusion/exclusion criteria, and informed consent was obtained from all participants.

### Participants

Inclusion criteria were: aged 65 or older, without cognitive impairment, living in nursing homes over the province of Albacete, whose management teams agreed to participate in the survey. The exclusion criteria were: 1, to stay in a temporary living situation or to live in the institution for less than three months; 2, to receive supplements or tube-feeding; 3, to suffer from an acute disease at the beginning of the survey; 4, to be in a terminal condition of health; and, 5, not to accept to take part in the survey. Nursing homes’ healthcare teams diagnosed acute diseases, terminal conditions and cognitive impairment according to specific clinical criteria for each resident (ICD-10 and DSM-IV-TR). Detailed information about inclusion and exclusion criteria is provided in S1 Fig. A total of 549 residents out of the 1460 residents meeting inclusion criteria were excluded due to the aforementioned exclusion categories. Therefore, a total of 911 individuals were screened and after a careful review and adjustment, 895 surveys constituted the final sample.

### Outcomes

The dependent variable, functional disability, was assessed by the Barthel Index (BI), a very useful tool designed to provide a rapid assessment of functional ability, easy to perform and widely studied and used across all geriatric levels [41]. The BI is an ordinal scale designed to measure performance in ten ADL functions: personal hygiene, bathing self, feeding, toilet use, stairs climbing, dressing, bowel control, bladder control, ambulation and chair/bed transfers. Three out of the ten items score a maximum of 5 (0 or 5 points), two items score a maximum of 15 (scores 0, 5, 10 or 15 points) and the remaining items score a maximum of 10 (0, 5 or 10 points). The total score ranges from 0 to 100 and classifies individuals from greater to lower level of dependence: 0–20 total, 21–60 severe, 61–90 moderate, 91–99 slight and 100 indicates independence. The BI was first developed and published in 1965 by Mahoney & Barthel [42] and some modifications have been published afterwards. In this survey the version developed by Shah et al. [43] has been used. Unlike the original BI, all items in this version have an equal number of categories: 0, 1, 3, 4 or 5 points for items scoring a maximum of 5 points; 0, 2, 5, 8 or 10 points for items scoring a maximum of 10 points; and 0, 3, 8, 12 or 15 points for items scoring a maximum of 15 points. In the original BI, the score assigned is based on the amount of physical assistance required, and the weighting of that item. In the modified scoring system, the score continues to depend on the weighting attached to the items. This modification improves sensitivity with respect to the original version and does not add difficulties when it is applied [43].

Given that functional disability may depend not only on the individual characteristics of the residents but also on the facility characteristics, the studied independent variables were classified in two levels: Level 1 comprised individual characteristics, and Level 2 included selected nursing home features.

A previous study using multivariate analysis considering only individual characteristics did not report significant association between BI and potential factors such as body mass index, prescription drugs, protein intake, acute diseases or psychological stress [44]. Taking into account these previous results, in this study, the following individual factors were considered in level 1: age, sex, self-perception of health status and mid-arm circumference. Nursing home features included in level 2 were: capacity (number of beds), type of funding (public or private), management (religious or secular) and location (main city or town/villages). These variables have been considered in different studies as potential factors influencing disability and other health problems [38,45].

### Statistical analyses

Considering both levels of data, we resorted to a multilevel analysis in order to take different sources of variability in the data. Multilevel models are regression models that include not only fixed effects related to individual and institution, but also random effects that allow for specific institution-based changes. Random effects can be also regarded as terms that model other unobserved variables that can have an effect on the BI. The general model and the four models that we considered are described in S2 Fig.

In order to compare the different models we resorted to Akaike’s An Information Criterion (AIC), the Bayesian Information Criterion (BIC) and conditional AIC (cAIC) [46]. AIC and BIC are widely used criteria that combines goodness-of-it, as measured by the likelihood of the fitted model, and penalizes more complex models. They are defined as -2*log(likelihood) + K*p, where *p* represents the effective number of parameters in the model and *K* is a penalizing constant that is 2 for the AIC and log-number of observations for the BIC. Models with lower values of the AIC or BIC are preferred. In all cases, the effective number of parameters *p* is the number of fixed effects (including the intercept) plus the number of variances. Significance of the fixed effects was assessed by comparing the corresponding t-value to a Student’s t with degrees of freedom equal to the degrees of freedom of the residual. Given the large number of degrees of freedom quantiles are very similar to those of a standard normal distribution; this distribution can be used when assessing significance.

The cAIC (conditional AIC) is similar to the AIC and BIC but it also consider the random effects when computing the complexity of the model and penalizing the model. Hence, it is a better suited model selection criterion for multilevel models, such as the ones we are using now, than the AIC or the BIC. Model complexity is measured by the *degrees of freedom*, with higher values indicating more complex models and the penalty term in the cAIC is twice the degrees of freedom. Hence, the cAIC should be considered as a more suitable model selection criterion for multilevel models than AIC or BIC.

Model fitting was done with package lme4 version 1.1–10 for the R programming language [47]. All models were fitted using maximum-likelihood estimation. We included study database in S1 Dataset.

### Ethical approval

The study was conducted in accordance with the principles articulated in the WMA Declaration of Helsinki (Ethical Principles for Medical Research Involving Human Subjects) [48]. The University of Castilla-La Mancha and the authorities for the Social Welfare in the province of Albacete approved the study. The Chair of the Office of the Delegate of Social Welfare in the province of Albacete offered help and assistance. We contacted to the manager of each institution by phone or e-mail and made an appointment in order to present our project and to obtain the permission to collect data in the institutions. The consent was obtained in accordance with local protocols. Given that individuals met inclusion criteria, no resident meeting these criteria suffered from cognitive impairments or any other problem that could affect the capacity of giving consent to participate in the survey. This was assessed by the nursing homes’ healthcare teams according to clinical criteria.

Residents were solely identified by a numerical code, both on the data collection form and in the computerized database. We followed Spanish Personal Data Protection Act [49] and Biomedical Research Act [50]. Prior to data collection the researchers obtained the oral consent of the residents after explain the study and answer questions. When required by the institution this oral consent was recorded using an audio recorder, or additional written consent was signed. Researchers provided safe custody of this information and they were the only ones who accessed to it. The researchers declare that no invasive procedures were involved in the study. The only information obtained from each resident was the required by the Barthel Index and variables of the study. Ethical approval was obtained from the Ethics Review Committee from Albacete.

Data collection was performed on the basis of these ethical principles. In the surveys, each resident was identified by a numerical code rather than by their first name, full mane or any other personal information. Age (and not birth date) was included in the surveys. For each resident, the database included the code plus age, gender, institution (also identified by a code) and clinical data obtained from the interview. These data were analyzed by the researchers.

## Results

The total sample was formed by 523 women (58.4%) and 372 men (41.6%). Women’s mean age was 82.9 (SD: 6.7) while men’s mean age was 81.3 (SD: 7.6). The mean age of the total sample was 82.3 (SD: 7.1). Table 1 shows the distribution of the elderly population participating in the survey and the distribution of BI scores among the different groups.

**Table 1:** Distribution of the elderly population participating in the survey and distribution of BI scores.

	Institutions	Distribution of the sample				BI^b^ scores		
	n	n	%	Women	Men	Mean	SD	95% CI
**Total sample**	34	895	100	-	-	76.7	26.5	74.9–78.4
**Sex:**								
**Women**	-	523	58.4	523	-	73.4	26.5	71.2–75.7
**Men**	-	372	41.6	-	372	81.2	25.9	78.6–83.9
**Age:**								
**65–69**	-	59	6.6	24	35	85.5	21.0	80.0–91.0
**70–74**	-	81	9.0	40	41	82.5	23.0	77.4–87.5
**75–79**	-	136	15.2	79	57	77.9	28.2	73.1–82.7
**80–84**	-	244	27.3	145	99	76.3	26.0	73.0–79.6
**85–89**	-	247	27.6	148	99	74.1	27.6	70.7–77.6
**≥90**	-	128	14.3	87	41	73.2	26.9	68.5–77.9
**MAC**^**a**^								
**<21**	-	29	3.2	18	11	66.4	29.2	55.3–77.5
**21–22**	-	60	6.7	32	28	69.4	30.2	61.6–77.2
**>22**	-	806	90.1	473	333	77.6	26.0	75.8–79.4
**SPH**^**c**^								
**Not as good**	-	74	8.3	50	24	65.7	28.9	59.0–72.4
**Does not know**	-	102	11.4	61	41	72.8	26.9	67.5–78.0
**As good**	-	330	36.9	184	146	73.7	28.2	70.6–76.7
**Better**	-	389	43.4	228	161	82.3	23.1	80.0–84.6
**Funding:**								
**Public**	12	428	47.8	224	204	81.9	24.0	79.6–84.2
**Private**	22	467	52.	299	168	71.9	27.8	69.3–74.4
**Management:**								
**Religious**	9	185	20.7	124	61	78.6	26.0	74.9–82.4
**Secular**	25	710	79.3	399	311	76.2	26.6	74.2–78.1
**Capacity (beds):**								
**<50**	12	171	19.1	108	63	76.0	22.0	72.6–79.3
**50–99**	10	179	20.0	99	80	74.3	28.4	70.1–78.4
**100–149**	4	156	17.4	100	56	79.6	27.3	75.3–83.9
**150–199**	4	87	9.7	42	45	80.2	22.6	75.3–85.0
**≥200**	4	302	33.7	174	128	75.9	28.2	72.8–79.1
**Location:**								
**Main city**	11	486	54.3	293	193	77.2	27.7	74.8–79.7
**Towns/Villages**	23	409	45.7	230	179	76.0	25.1	73.6–78.4

^a^ MAC: Mid-arm circumference

^b^ BI: Barthel Index

^c^ SPH: Self-perception of health in comparison with others of the same age

The mean BI score for the total sample was 76.7 (SD: 26.5) points. BI classified residents as follows: 5.8% (n = 52) totally dependent, 17.9% (n = 160) severely dependent, 33.4% (n = 299) moderately dependent, 22.7% (n = 203) slightly dependent and 20.2% (n = 181) independent subjects. Table 2 summarizes the results for the four models designed for the multilevel examination.

**Table 2:** Results for the four multilevel models fitted to the data in the study.

Variables	Model 1^a^^,^^b^	Model 2 ^a^^,^^b^	Model 3 ^a^^,^^b^	Model 4 ^a^^,^^b^
**Intercept**	76.337 (1.838)^h^	104.948 (10.738) ^h^	71.196 (6.445)^h^	100.449 (12.273)^h^
**Age**		-0.569 (0.114)^h^		-0.562 (0.114)^h^
**Sex (reference female)** **Male**		6.171 (1.642)^h^		6.139 (1.644)^h^
**SPH**^**c**^ **(reference worse)** **Does not know** **As good (equal)** **Better**		6.976 (3.677) 6.494 (3.080)^g^ 16.851 (3.039)^h^		7.224 (3.675)^g^ 6.757 (3.082)^g^ 16.967 (3.038)^h^
**MAC**^**d**^ **(reference <21)** **21–22** **> 22**		3.257 (5.503) 5.251 (4.478)		3.528 (5.509) 5.087 (4.478)
**Funding (reference private)** **Public**			9.766 (3.917)^g^	7.939 (3.923)^g^
**Management (reference secular)** **Religious**			7.615 (4.364)	7.391 (4.330)
**Capacity**			-0.007 (0.032)	-0.006 (0.032)
**Location (reference main city)** **Towns/Villages**			0.137 (4.497)	-0.690 (4.463)
**Sigma**^**2**^ **error**	596.58	541.04	597.12	541.38
**Sigma**^**2**^ **random effect**	81.27	77.41	60.53	62.14
**Degrees of freedom of the residual**	892	885	888	881
**AIC/BIC**^**e**^	8312.0/8326.4	8239.7/8287.7	8314.0/8347.5	8243.1/8310.2
**cAIC (d.f.)**^**f**^	8283.1 (23.6)	8210.9 (31.4)	8283.6 (22.4)	8210.7 (30.2)
**Deviance**	8306.0	8219.7	8300.0	8215.1

^a^ For the covariates, values indicate point estimates of their coefficients, with standard errors in parentheses.

^b^ For the error and random effect, the values indicate point estimates of their respective variances.

^c^ SPH: Self-perception of health in comparison with others of the same age

^d^ MAC: Mid arm circumference

^e^ AIC/BIC: Akaike’s An Information Criterion / Bayesian Information Criterion

^**f**^ cAIC (d.f.): conditional Akaike’s An Information Criterion (degrees of freedom)

^g^ P < 0.05

^h^ P < 0.001

Model 1 sets the baseline for all the other models. When individual covariates are introduced in Model 2, the resulting model showed that age, sex and self-perception on health status are highly significant. As people get older their BI decreases. Male tend to have a better BI than female. Finally, we want to highlight that self-perception of health status is also significant. In particular, people that feel they have a better health reach higher BI scores. The effects of institution level covariates are shown in Model 3. Only institution ownership is significant, with state-owned institutions providing a higher BI than privately-owned institutions. Model 4 is a full model that accounts for individual and institutional effects. It should be noted that all variables have very similar coefficient estimates and standard errors when compared to Model 2 and Model 3, with the same level of significance. Self-perception at ‘equal’ level becomes mildly significant now. The fact that coefficient estimates and standard errors of the covariates do not change means that they clearly explain different levels of variation in the data and that there is no interaction among them.

As seen in Table 2, Model 2 showed the lowest AIC and BIC. Model 4 showed the lowest deviance and cAIC, and Model 2 had a slightly higher value of the cAIC and a higher complexity (i.e., higher value of the degrees of freedom). As mentioned earlier, AIC and BIC do not penalize for the complexity due to the random effects, as the cAIC does. Hence, the cAIC is a better criterion for model selection in this case.

After considering the number of significant variables, the values of the deviance, the values of the different model selection criteria and the fact that Model 4 has an institution level significant variable and a lower variance of the random effects (which means lower unexplained differences among institutions), it seems that Model 4 provides the best fit to the data without being extremely complex.

Finally, the use of multilevel models with random effects has the advantage of providing estimates of the effect of the residence on BI that has not been measured by the observed covariates. This is provided by the random effect estimate for each institution, and it can be regarded as the residual effect of the institution, that in most cases shows the effect of unmeasured covariates. This illustrates the differences among the residuals effects of the nursing homes. Multilevel modeling has also the ability to provide estimates of the institutional effect isolated from the residents’ effects. For this, we can simply focus on the part of the model related to the institutional effects.

S3 and S4 Figs show ranked point estimates of the institution total effect together with an interval that covers plus/minus one standard error and the mean BI of the residents for each nursing home. Some of the institutions with high mean BI values show a poor institutional effect when the residents’ variables were taken into account. This highlights the importance of separating individual and institutional effects when evaluating the quality of the nursing homes.

## Discussion

Our results showed that older age, female gender, living in private nursing home, and negative self-perception of health are factors associated with high level of ADL dependence, among residents without cognitive impairment in Spain.

Increasing age is associated with increasing frailty and dependence. Functional impairment is one of the most important determinants of dependence in elderly. The average life expectancy is increasing and a greater portion of older people live in their own home as long as possible, which is conditioned by the ability to live an active life. When dependence levels are high, they usually enter in an institution until the end of their lives [26].

Individual factors and nursing home features influence residents’ functional ability. The ability to perform ADL functions and other vital goals are involved in health concept. Although health status is closely related to the presence or absence of illness and dependence, it is associated with subjective factors such as self-perceived health. In this way, a poor self-perception of health has been found to be influent in dependence [20,21], so it is common to be included in medical and social research [51,52].

Prevalence of disability among older people is high in developed countries and it is increasing due to the sustained grow in longevity [20,53]. Among the indicators of functional impairment, ADL disability is one of the most commonly used worldwide and BI is the most used scale to measure ADL limitations as part of the Comprehensive Geriatric Assessment [41]. Our sample showed a high prevalence of functional impairment (near to 80%), according to BI. This percentage is similar to the results found by Onder et al [54] in a study performed in 57 nursing homes of seven European countries, and is slightly lower to the findings published by Nakazawa et al. [55] in 140 nursing homes in Japan. Levels of ADL dependence are higher in institutionalized elderly when are compared to results in community dwelling older people [53] and also more higher prevalence rates are commonly found in nursing homes [54]. This could be explained, at least in part, by the fact that older people are quite often institutionalized when disability is high that it is not possible to take care of them at home. In fact, data provided by the Department of Health and Social Welfare of Castilla-La Mancha (Spain) indicate that only 10% of dependent people were living at home in 2012. The fact that our population has shown lower levels of disability than other studies may be explained by the characteristics of our sample which did not include individuals with cognitive impairment and it is well known the association between cognitive impairment and disability [22].

In our study gender, age, self-perception of health, and institution ownership were found to be associated with functional status. Regarding the role of ageing and gender on functional ability, while our findings confirm the widely demonstrated trend that independence in ADL declines with increasing age especially in the oldest old people [6,19–21,56], women showed higher prevalence of ADL disability as compared with men, especially in the older categories, without agreement about gender differences found in the literature when institutionalized elderly has been studied [19,53,57].

Self-perceived health includes the individuals’ internal and external resources and their perception of their physical and psychological health. Although some studies have found a decrease in the number of older people who perceived themselves healthy when chronic diseases and degree of disability are increased [58], most of them support that older people perceive themselves as healthy [52]. A negative self-perception of health has been found to influence dependence [21], and a poor perception of ageing has been shown as an indicator of future dependence in ADL [59]. In line with this observation, our study showed significant association between positive self-perceived health and high levels of independence in ADL. We found that more than 40% of elders felt healthy when compared with other people of the same age. Changes in health due to age are influenced by social norms. Disabilities and health problems can be seen as normal for older people and may be ignored. The system may not help disabled individuals to have the ability to adapt to their new status. A positive attitude to health can help individuals to accept symptoms of illness as a part of their health. Adaptation is a process, by which someone adjusts to and prepares for changes in life. From this point of view, it is important to maintain good self-perceived health in the elderly as far as possible due to it will be easier to promote health among them.

Not only physical and psychological conditions but also environmental, socioeconomic and lifestyle factors may influence functional status of elder people. In fact, socioeconomic and lifestyle aspects appear to have major influence on functional status of elders living in the community [60]. On the other hand, institutionalized older people environment includes the institution features and it is important to study the relationships of these features with residents’ dependence. Among the key nursing home characteristics selected in our study, only ownership showed significant differences. Better functional status was found in individuals living in public (non for profit institution) nursing homes than subjects living in private ones. These results may be explained, at least in part, by the fact that all the places available in public residences are occupied and an important number of elders should wait for the allocation of a place in a public nursing home. In these cases, the alternative is to entry into a private nursing home or the family takes older care [31]. During the waiting period, older people delay their nursing home admission. But finally, elders must enter in a nursing home with a functional state worsened, because this delaying in the entrance [31]. Another possible explanation is that some private residences in Albacete could apply more paternalistic care models [61,62], where institutions assumed that caregivers would know what is best for the resident, provoking effects on autonomy and functional capacity of the residents [61,62]. Previous studies have shown that the care provision style of institutions is represented by institutional policies and organization may promote nursing home residents’ quality of life and functional status [61]. Certain values are assumed as central for quality of care, including promotion of self-determination, individualized care and nursing home organization issues, although they do not necessarily meet resident’s preferences and needs [33]. Today in Spain, public nursing homes try to be more like a home, a place where the residents are in control and make their own decisions about their care whenever possible. They structure their internal organization in an attempt to minimize as much as possible its effect on the each resident’s ADL [33,35]

Our study did not find an association between functional decline and nursing home features such as capacity, location or management (religious and/or secular). Residents living in religious nursing homes are encouraged to participate in different religious activities, and it has been found that more frequent attendance at religious services is associated with lower levels of functional disability [63,64]. However, there is a complex association between religious activities and functional limitations in older adults. Other studies found that receiving more tangible support from religious congregations was associated with more functional limitations measured contemporaneously, but were associated over time with a less rapid worsening of disability [65]. Attendance to religious activities it is not compulsory in our religious nursing homes. In fact, many residents are not religious practitioners even they are not religious believers. This fact may explain, at least in part, our findings.

Although advantages and disadvantages of living in a “small” or “large” nursing home have been discussed, there are few studies addressing the impact of the size or the location of the institution on functional status. While it has been found that the number of beds of the institution is significantly associated with change in functional status of residents [66], we did not find an association between ADL limitations and institution size in our sample. As we previously commented, today it is a challenge how to provide residents the sense of home in a nursing home [67]. Trying to live in the same manner as one always had and interaction with family and friends are factors influencing the sense of home. Nursing homes located in large or very large cities have higher proportion of residents not feeling at home in the nursing home [67]. In our study the main city and also the villages do not exceed 200,000 inhabitants, therefore the proximity of the residents to their relatives is assured.

Other nursing home characteristics such as safety features, staffing ratios, services, or support provided could also influence residents’ functional status [11,12,16]. In Spain, current regulatory framework [68] establishes a set of minimum conditions on the staff and safety standards applicable in all institutions which are strictly fulfilled by all nursing homes. In this sense, it is interesting to note that these regulations envisage more staffing ratios for disabled residents (one nurse for every 50 independent residents and one nurse every 40 dependent residents). As commented previously, our multilevel models include random effects that account for any unobserved variables that can have an effect on the BI.

The 34 studied nursing homes showed significant differences according to their institutional effects. Multilevel models can help separate individual from institutional effects that can lead to a more accurate evaluation of the institutional performance. Doing further research in nursing homes showing worse results can help improve the quality of the institution.

Among the strengths of the study, we can include the large sample of participants, not only elderly people but also nursing homes all over the province of Albacete. Another strength is the homogeneity in data collection because all the tests were completed by only three trained nurses. On the other hand, the influence of nursing home features on different aspects of disability is a topic largely unexplored and few studies have examined nursing home features such as management, type of funding, location or capacity as potential factors associated with residents’ ADL disability. To the best of the authors’ knowledge, this is the first study performed in Spain including individual and nursing home characteristics as potential factors influencing ADL disability. However, we should recognize some limitations. Our sample did not include cognitive impaired elderly. On the one hand, data collection process was performed by interviewing the residents and on the other hand, it is known that effects of nursing homes on ADLs tend to be weaker for residents with low cognitive function [69]. Moreover, residents without cognitive impairment is a target group that may benefit from programs that have a different structure than others designed for residents with dementia [70]. Therefore, research including this population group would be welcomed. On the other hand, a larger number of measures could provide best fitted models.

## Conclusions

The current study found high levels of ADL dependence among institutionalized elders. Gender, age, self-perception of health, and institution ownership were associated with functional status. Additionally, significant differences among institutions were found. Disentangling individual and institutional effects by means of multilevel models can help evaluate the quality of the residences.

## Supporting information

S1 Dataset Study database. (XLSX)

S1 Fig Flow diagram of included/excluded participants and data collection. (TIFF)

S2 Fig Multilevel models including individual or/and institutional variables. (TIFF)

S3 Fig Ranked point estimates of the institutional total effect together with an interval that covers plus/minus one standard error. (TIFF)

S4 Fig Average Barthel Index of the residents for each residence plus a horizontal line with the global average Barthel Index. (TIFF)
