# Supplementary figures and images for: Individual and institutional factors associated with functional disability in nursing home residents: An observational study with multilevel analysis
Source: PLoS One. 2017 Aug 28;12(8):e0183945. doi: 10.1371/journal.pone.0183945 (PMC5573540; doi:10.1371/journal.pone.0183945)

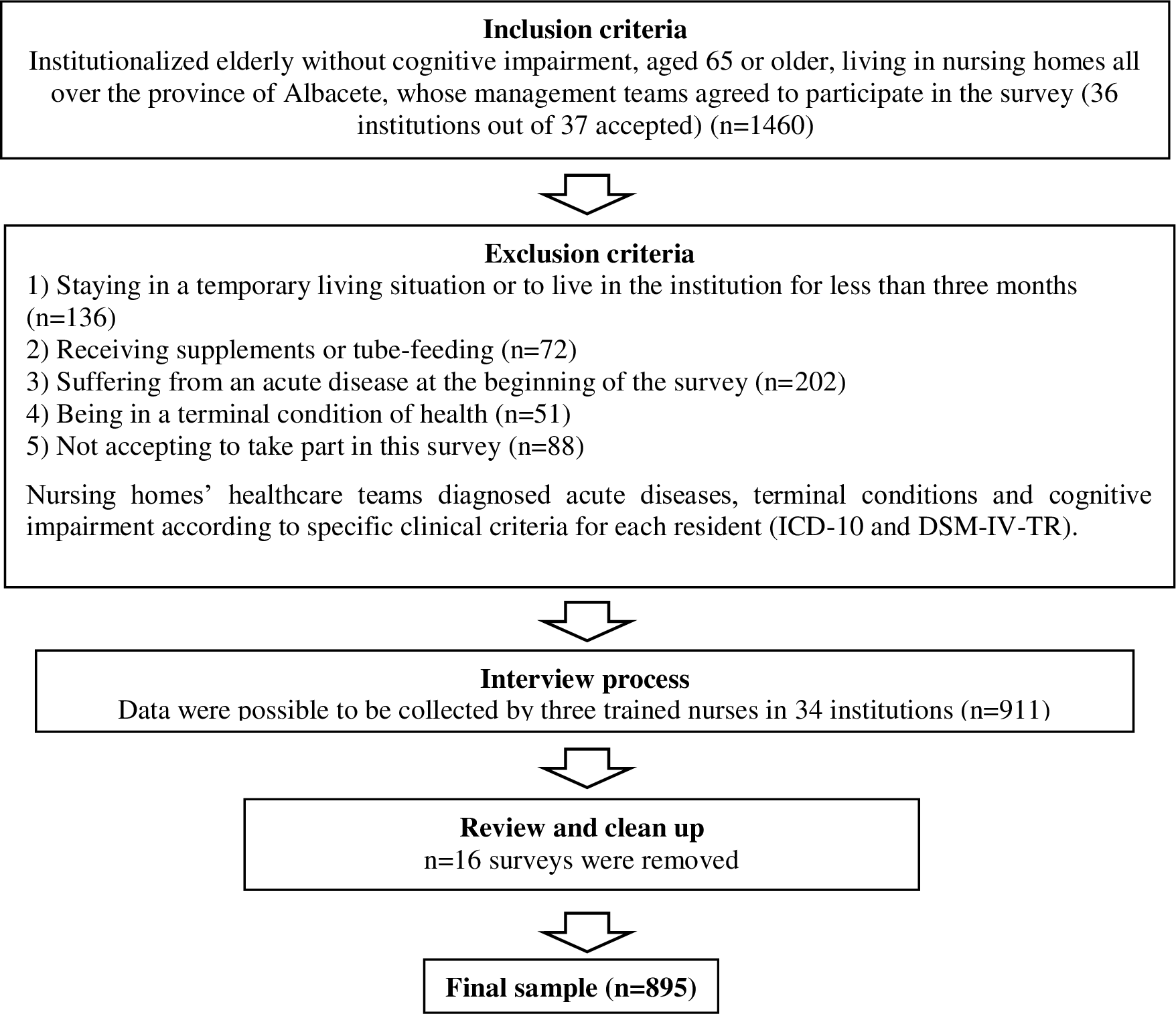

Supplement: S1 Fig — (TIFF) [file pone.0183945.s002.tiff]

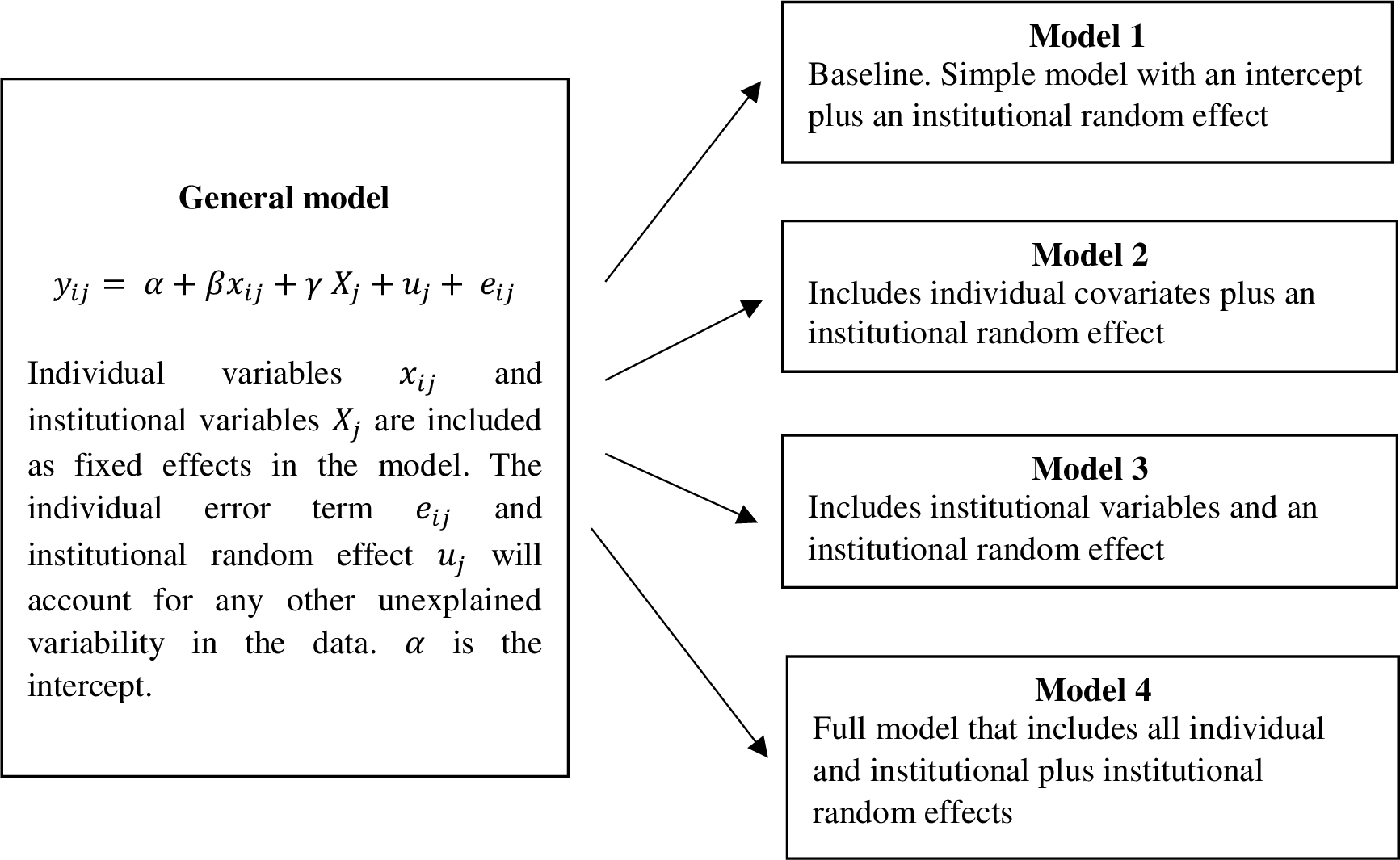

Supplement: S2 Fig — (TIFF) [file pone.0183945.s003.tiff]

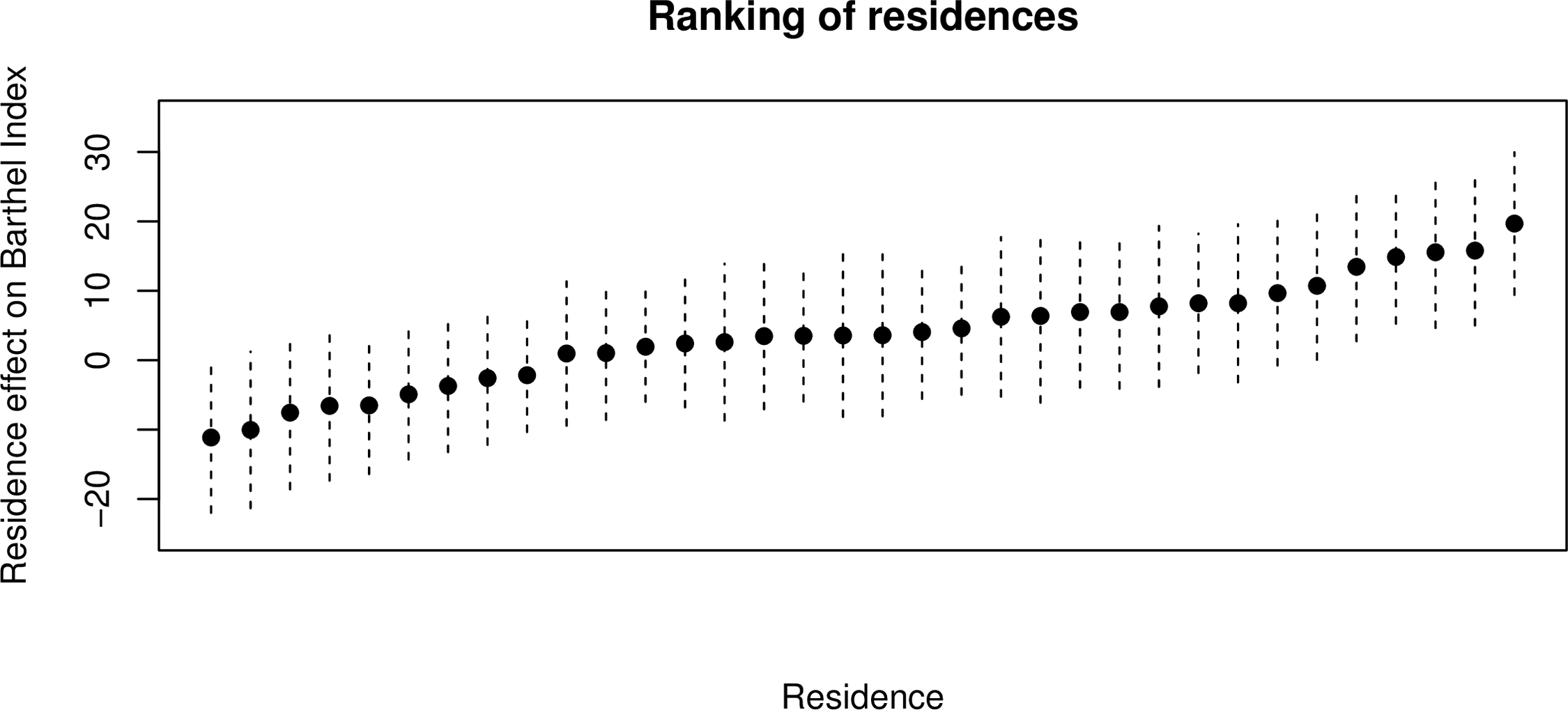

Supplement: S3 Fig — (TIFF) [file pone.0183945.s004.tiff]

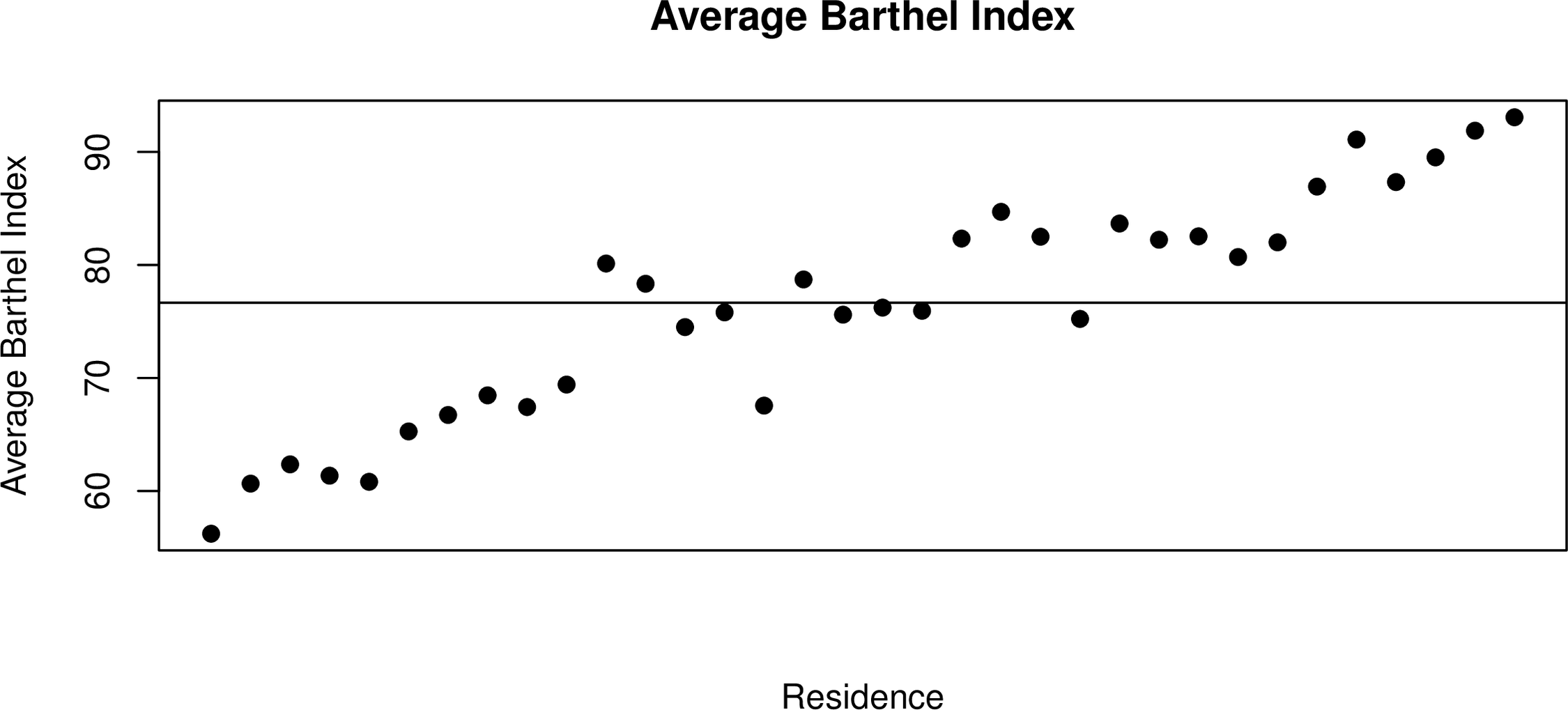

Supplement: S4 Fig — (TIFF) [file pone.0183945.s005.tiff]
